# Passive Cushiony Biomechanics of Head Protection in Falling Geckos

**DOI:** 10.1155/2018/9857894

**Published:** 2018-02-19

**Authors:** Hao Wang, Wenbo Wang, Yi Song, Lei Cai, Zhendong Dai

**Affiliations:** ^1^College of Astronautics, Nanjing University of Aeronautics and Astronautics, Nanjing 210016, China; ^2^College of Aerospace Engineering, Nanjing University of Aeronautics and Astronautics, Nanjing 210016, China

## Abstract

*Gekko geckos* are capable to crawl on the steep even on upside-down surfaces. Such movement, especially at great altitude, puts them at high risks of incidentally dropping down and inevitable body or head impactions, though they may trigger air-righting reaction (ARR) to attenuate the landing shocks. However, the air-righting ability (ARA) in *Gekko geckos* is not fully developed. The implementation of ARR in some geckos is quite slow; and for those without tails, the ARR is even unobservable. Since ARA is compromised in *Gekko geckos*, there must be some other mechanisms responsible for protecting them from head injuries during falls. In this study, we looked into a *Gekko gecko*'s brain to study its internal environment and structure, using the magnetic resonance imaging (MRI) technique. The results showed that the brain parenchyma was fully surrounded by the cerebrospinal fluid (CSF) in the skull. A succulent characteristic was presented, which meant the intracalvarium was significantly occupied by the CSF, up to 45% in volume. Then a simplified three-dimensional finite element model was built, and a dynamic simulation was conducted to evaluate the mechanical property of this succulent characteristic during the head impactions. These implied the succulent characteristic may play certain roles on the self-protection in case of head impaction, which is adaptable to the *Gekko gecko*'s locomotion and behavior.

## 1. Introduction

Many animals in nature are able to crawl, climb, or run on different inclined surfaces, such as walls, ceilings, branches, and leaves, at a certain height above the earth. Falling down, as a common experience, is somehow unavoidable. To avoid the body being injured when falling and impacting with the ground, animals have developed various abilities, such as air-righting abilities (ARA). Cats try to make their feet to attach on the ground at first to reduce the impact forces acting on the body by turning up the body upside-down through the vertebra and tail [1–4]. *Gekko geckos* turn up by rotating their tail [5]. Preventing the brain injury from the falling and impacting to the ground, a sort of self-protection is one of the important survival skills gained from natural selection and evolution in animals. Aerial maneuverability or air-righting performance is the most important mechanism of self-protection in insects [6, 7], cats [1–4], rats [8, 9], rabbits [10], frogs [11], and geckos [5]. However, the air-righting is not always performed perfectly in geckos. It was showed that the geckos without tails could not perform air-righting reaction (ARR) at all, and even for the geckos with tails, almost one-tenth could not perform ARR well [5]. Actually, the ARA might be weaker in *Gekko geckos*, because the relevant air-righting reflex did not mature, developmentally speaking, into a complete central program. The published data [9] provided evidence that the ARA probably starts developmentally as a reflex and within days/weeks mature into a central pattern generator (CPG) by showing that the completion of the ARR maturation process had no dependency on loads attached to different parts of the body. In our behavior experiments, not all geckos could turn around successfully in abdomen-up falling down. Especially when the falling height is not so high, the time for free fall is not sufficient for this performance. Even though, it rarely causes any injury in the brain. The head impaction is cushioned somehow. It seems that *Gekko geckos* may possess certain characteristics to prevent themselves especially their head from injury caused by impacting the ground while falling down. To disclose the potential mechanism underlying the self-protection in head impaction, it is necessary to look into the intracalvarium structure and material of the animal's head.

Not like a woodpecker's head, which has been studied thoroughly for decades [12–15], a *Gekko gecko*'s head has been rarely investigated, since its significance of antishock characteristic is much lower than the former. The woodpecker's head can stand high-frequency shocks that are introduced from its drumming beak during the daily forage, while the gecko's head is only in the risk of one sudden head shock caused by an incident dropping. The underlying mechanism of head protection should be different. Here, we took a close look at the intracranial structures by the magnetic resonance imaging (MRI) technique. Then a simplified mathematical model was built to qualitatively evaluate the corresponding mechanical property.

## 2. Materials and Methods

### 2.1. Experimental Animals

The *Gekko gecko* lizards were brought from Nanning, Guangxi Province, China, and habituated to the study colony for two months before the experiments. The mean temperature and relative humidity were 25°C and 65%, respectively, which were close to the values for the natural ambiance of *Gekko geckos*. Adult *Gekko geckos* weighted 40–70 g were selected for the MRI study.

The entire study was carried out in accordance with the Guide of Laboratory Animal Management Ordinance of China and approved by the Jiangsu Association for Laboratory Animal Science (Jiangsu, China).

### 2.2. The Intracalvarium Morphology Investigation

The intracalvarium morphology was investigated by two ways, the qualitative observation after the surgical anatomy and the quantitative measurement using MRI.

**Figure 1:**
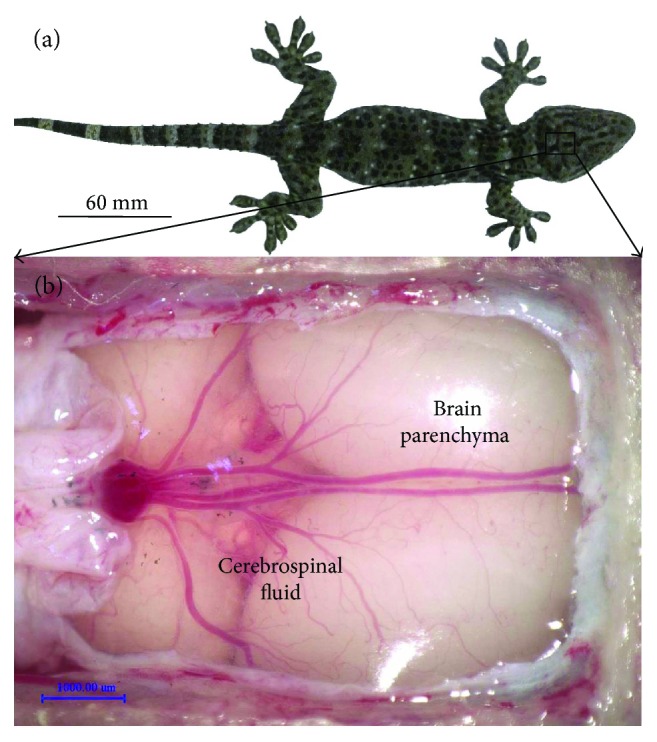
(a) A *Gekko gecko* lizard and (b) the observation on the intracalvarium of gecko brain using a digital microscope (VHX-600, Keyence, Japan) after craniotomy (the skull was opened and the dura was removed while the arachnoid was intact).

After the surgical anatomy, we found that the gecko's brain parenchyma (Figure 1) is surrounded by the cerebrospinal fluid (CSF).

**Figure 2:**
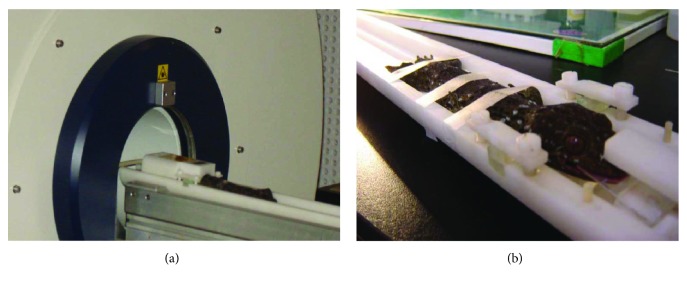
An anaesthetized gecko is placed into the MRI instrument, whose head and body are fixed to a custom-designed fixture (a). The close view of the fixed gecko in the fixture without cover (b).

**Figure 3:**
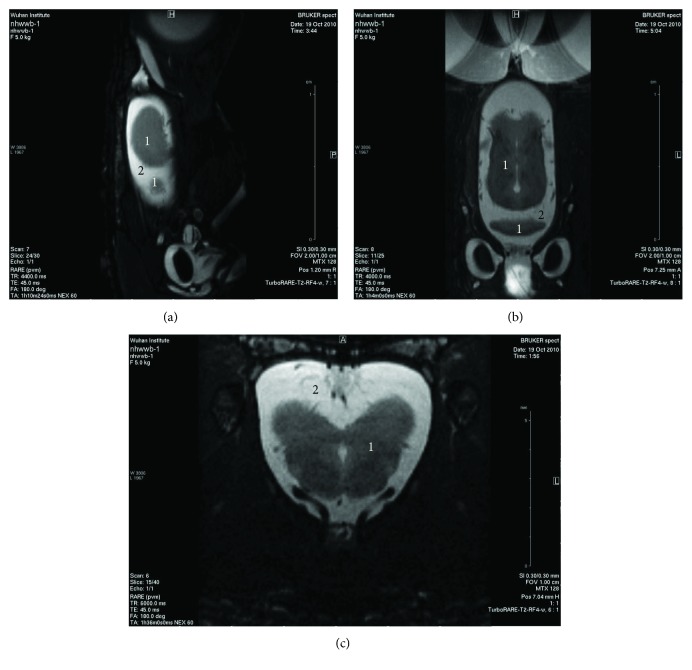
The MRI scanning of a gecko head in the sagittal plane (a), horizontal plane (b), and coronal plane (c). The dark area (marked by “1”) indicates the brain parenchyma while the bright area (marked by “2”) the CSF.

For MRI investigation, the animal was anaesthetized by the intraperitoneal injection of 0.4% sodium pentobarbital in a 0.75% NaCl solution. A dose of 0.75 ml/100 g body weight was administered. After the pain reflex had disappeared, the gecko was fixed to a custom-designed fixture (manuscript in preparation, see Figure 2) and then placed into the MRI instrument (BioSpec 7T/20 cm, Bruker, Germany). The whole brain was scanned in three orthogonal (sagittal, coronal, and horizontal) planes (Figure 3), and the corresponding spatial interval of the scan was all 0.30 mm.

Based on the MRI image sequence, the distribution and the volume of the CSF in the skull were evaluated with the help of the open source software ImageJ (http://rsbweb.nih.gov/ij/). It is not difficult to distinguish the brain parenchyma and the CSF by gray level of the MRI image. Then, the surface integral and the volume integral were conducted for the brain parenchyma and the CSF, respectively.

## 3. Results and Discussion

### 3.1. Distribution of the CSF in the Gecko Skull

**Figure 4:**
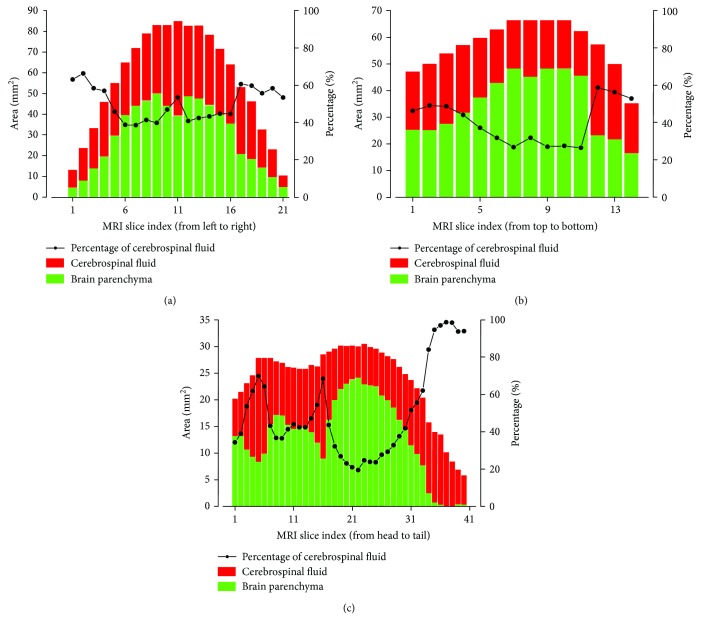
The percentage distribution of the CSF in the gecko skull along the coronal direction ((a), sagittal plane), vertical direction ((b), horizontal plane), and sagittal direction ((c), coronal plane). The outer envelope of the CSF indicates the full sectional area of the inner skull.

The percentage distribution of the CSF in the gecko skull is shown in Figure 4. Since the dimensions of the brain along the sagittal, coronal, and vertical directions are different, the number of slices is variable. It provided relatively more detail along the sagittal direction (coronal plane, Figure 4(c)), so the corresponding image sequence was employed to calculate the volume of the brain parenchyma and the CSF. The distribution is clearly symmetrical along the coronal direction (sagittal plane, Figure 4(a)) due to the morphologically bilateral symmetry of the brain. The MRI images and the diagrams show that the brain parenchyma is fully surrounded by the CSF. The minimum percentage of the CSF is still above 20%.

Based on the data shown in Figure 4(c), the volume of the brain parenchyma and the CSF was integrated as 159.0 mm^3^ and 127.4 mm^3^, respectively. Thus, the CSF accounts for about 45% of the entire brain volume.

### 3.2. The Finite Element Model of a Simplified Gecko Brain

**Figure 5:**
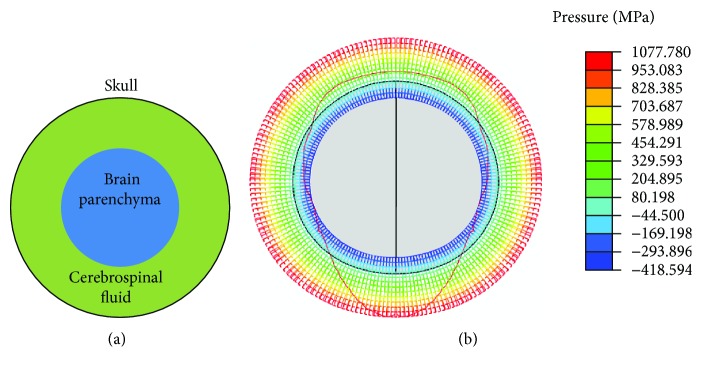
(a) The simple finite element model of a gecko brain. (b) The pressure distribution around the brain, indicated by a red curve. The black circle denotes the brain surface, and the color-coded mesh represents the values of pressure.

The gecko brain was simplified into concentric spheres filled with liquid interval (Figure 5(a)). The parenchyma density of the gecko brain was around 1060 kg/m^3^, which was estimated by the ratio of mass to volume. Considering the irregular geometrical shape, the parenchyma volume was decided using the fluid volume measuring method. The density of the brain parenchyma [16, 17] is quite close to 1036 kg/m^3^. The density of CSF was also measured around 989 kg/m^3^. And the viscosity coefficient (*μ*) of the CSF [18] is around 0.85 mPa·s.

The plan model was introduced to present the mechanics of the head impact cushioning. The contact target surface (ground) is defined as a rigid body in the model. The elasticity modulus and passion ratio of skull and brain parenchyma are set to be 15 GPa and 0.3 GPa, respectively. The Young's modulus for the CSF was set at 2.436 GPa, similar processing as the biomechanical study on the hemolymph in insects [19].

The finite element model was built and simulated using the ABAQUS/Explicit (ABAQUS 6.6, ABAQUS, Inc., USA), which is always chosen to solve nonlinear dynamical problems such as impact and explosion. Considering the symmetrical structure, the finite element model was built as a half-plane strain model. The initial contact between the ground and the skull, between the CSF and the skull, and the brain and the CSF were established using the contact-pair tool in ABAQUS/Explicit. All the structures were meshed into a quadrilateral, and the CPE4R elements were employed throughout. The model was built based on the fundamental principles of continuum mechanics, and the simplification on the fluid of CSF also had precedents [19], which guaranteed the model as secure as possible.

The simulation showed the pressure distribution around the brain was varying during the interaction between the head and the ground. When the two objects got stuck, strain built up. Along the vertical direction, the compressive stress was increasing that caused positive pressure on the brain, while along the lateral direction, the tensile stress was increasing that caused negative pressure on the brain (Figure 5(b)).

The CSF accounts for around 45% of a gecko brain. Different proportion of the fluid may affect the pressure limits caused by falling and head impact. Additionally, the height of falling could be another factor. Here, we simulated two proportions of the CSF, 45% and 22%, given two different falling heights, 1 m and 0.5 m, respectively. The comparison is shown in Table 1.

### 3.3. The Succulent Characteristic of a Gecko's Head

The MRI investigation has disclosed the succulent characteristic of a gecko's head. The intracalvarium of the head is full of the CSF, up to 45% in volume. Animal skulls contain a space between the brain and the skull's vascular tissue, called a subarachnoid cavity. The cavity houses the CSF. It is said that CSF can only provide cushioning from minor bumps and jostling. In the instances of strong vibrations or blows, CSF will allow excessive movement of the brain, potentially resulting in bruising and concussions. One factor of the antishock mechanism in the woodpecker is that it has relatively little CSF [12, 20], thereby reducing the transmission of the mechanical excitations into the brain through the CSF [21, 22]. This is contradictory to what we found in a gecko's head. However, from a physical point of view, the mode of head shock in a woodpecker is different from that in the gecko. The former is a bilateral vibration in a certain frequency, by which the brain parenchyma may be restrained near the equilibrium position relative to the skull. The latter is an instant unidirectional impaction, by which the brain parenchyma may overshoot far from its equilibrium position. This might be the reason why the amounts of CSF in the woodpecker and in gecko are so different.

According to the comparison in Table 1, the high proportion of the CSF helps to reduce the maximal positive pressure, around 16% and 13%, at the falling height 1 m and 0.5 m, respectively. But it also causes an increase in the maximal negative pressure, around 8% and 11%, at the falling height 1 m and 0.5 m, respectively. Both positive and negative pressures in the intracalvarium are the reasons for brain injury [23]. Decreased positive pressure and increased negative pressure are the two opposites of the self-protection in the head impaction but which affects the brain more is still unknown. Speculatively, especially for the high falling height (1.0 m), for the high proportion of the CSF, the benefit of decreasing positive pressure (16%) surpasses the perils of increasing negative pressure (8%). This may be one of the advantages of natural selection and evolution for animals who can move at high altitude.

The finite element model and simulation developed in here is quite simple, which need further investigation using more fidelity models, such as considering the complex cortical bone property [24, 25]. However, the simple model has implied the succulent characteristics of head impact in *Gekko geckos* qualitatively from several aspects. First, the fluid spreads the impulse across a wide area, allowing the material to absorb more of the impact. Second, the hydrodynamic drag gradually slows down the motion of the brain parenchyma caused by inertia after head impact. Thirdly, the good liquidity of the CSF attenuates the positive pressure in the intracalvarium. All those imply that the succulent characteristics of a gecko's head may play an important role in the self-protection in the head impaction, which is worth further studying and supplements to the behavioral and bionic application studies in *Gekko geckos* [26].

ARA is one of the important abilities for animals who move on high. It has been shown that ARA probably starts developmentally as a reflex and within days/weeks matures into CPG [9]. From an evolutionary point of view, this is a relatively later evolutionary state. The succulent characteristic of a gecko's head may provide a case in which the ARA starts in an earlier evolutionary state. More importantly, this may imply how nature has compensated for this by providing an alternative approach to protect the head. As evolution progressed, from reptiles to birds and mammals, the ARA developed and the need for 45% CSF was reduced, thus releasing space for the brain.

## 4. Conclusion

This study investigated the internal environment and structure of a *Gekko gecko*'s brain qualitatively and quantitatively, in order to understand their mechanism underlying the self-protection in head impaction. This study was also necessary in order to discover possible alternative mechanisms, besides the air-righting abilities, responsible for protecting animals moving on high from head injuries in falling. The succulent characteristic of a *Gekko gecko*'s brain was disclosed. By means of surgical anatomy, it was shown that the *Gekko gecko*'s brain parenchyma was fully surrounded by the CSF, while by means of the MRI techniques, it was shown that the intracalvarium was significantly occupied by the CSF, up to 45% in volume. The three-dimensional finite element model and its simulation on the impactions due to falling from different heights showed that the succulent characteristic contributed to the positive pressure decreasing during the head impaction but also to the negative pressure increasing. The former is beneficial to the self-protection in head impactions while the latter is not. Though the decreased positive pressure showed clear advantages over the increased negative pressure when falling from great height, at the point of view of the change rate in pressures, it needs further detail and elegant comparative studies to draw comprehensive conclusions. To our certain knowledge, to date, there has been no systematic interspecific or intraspecific comparative studies on how the CSF affects the mechanical property of the head and the consequence in self-protection. As one step further of this research, a fidelity finite element modeling study and physical model validation involving the morphology of the brain, the CSF and the skull, and their mechanical properties are demanded.

## Figures and Tables

**Table 1:** The comparison of pressure on the brain parenchyma for different proportion of the CSF at different falling heights.

Height (m)	CSF 45%		CSF 22%	
	Positive pressure (MPa)	Negative pressure (MPa)	Positive pressure (MPa)	Negative pressure (MPa)
1.0	1077.78	428.92	1285.06	396.25
0.5	925.93	337.26	1064.86	302.90

## References

[1] Kane T. R., Scher M. P. (1969). A dynamical explanation of the falling cat phenomenon. *International Journal of Solids and Structures*.

[2] Marey E. J. (1984). The movements that certain animals execute to fall on their feet when they are tossed from an elevated place (translated from French). *Science*.

[3] McDonald D. A. (1960). How does a cat fall on its feet?. *New Scientist*.

[4] Montgomery R. (1993). Gauge theory of the falling cat. Fields Institute. *Communications*.

[5] Jusufi A., Goldman D. I., Revzen S., Full R. J. (2008). Active tails enhance arboreal acrobatics in geckos. *Proceedings of the National Academy of Sciences of the United States of America*.

[6] Yanoviak S. P., Munk Y., Kaspari M. A., Dudley R. (2010). Aerial manoeuvrability in wingless gliding ants (*Cephalotes atratus*). *Proceedings of the Royal Society B*.

[7] Yanoviak S. P., Munk Y., Dudley R. (2011). Evolution and ecology of directed aerial descent in arboreal ants. *Integrative and Comparative Biology*.

[8] Laouris Y., Kalli-Laouri J., Schwartze P. (1990). The postnatal development of the air-righting reaction in albino rats. Quantitative analysis of normal development and the effect of preventing neck-torso and torso-pelvis rotations. *Behavioural Brain Research*.

[9] Laouris Y., Kalli-Laouri J., Schwartze P. (1990). The influence of altered head, thorax and pelvis mass on the postnatal development of the air-righting reaction in albino rats. *Behavioural Brain Research*.

[10] Schönfelder J. (1984). The development of air-righting reflex in postantal growing rabbits. *Behavioural Brain Research*.

[11] Wang H., Wang L., Shao J. D., Liu T. T., Dai Z. D. (2013). Long hindlimbs contribute to air-righting performance in falling tree frogs. *Journal of Mechanics in Medicine and Biology*.

[12] May P. R., Fuster J. M., Newman P., Hirschman A. (1976). Woodpeckers and head injury. *The Lancet*.

[13] May P. R., Fuster J. M., Haber J., Hirschman A. (1979). Woodpecker drilling behavior. An en-dorsement of the rotational theory of impact brain injury. *Archives of Neurology*.

[14] Stark R. D., Dodenhoff D. J., Johnson E. V. (1998). A quantitative analysis of woodpecker drumming. *Condor*.

[15] Zhu Z. D., Wu C. W., Zhang W. (2014). Frequency analysis and anti-shock mechanism of woodpecker’s head structure. *Journal of Bionic Engineering*.

[16] Hickling R. L., Wenner M. L. (1973). Mathematical model of a head subjected to an axisymmetric impact. *Journal of Biomechanics*.

[17] Dawson S. L., Charles S. H., Lucas F. V., Sebek B. A. (1980). The countrecoup phenomenon: reappraisal of a classic problem. *Human Pathology*.

[18] Bloomfield I. G., Johnston I. H., Bilston L. E. (1998). Effects of proteins, blood cells and glucose on the viscosity of cerebrospinal fluid. *Pediatric Neurosurgery*.

[19] Dai Z. D., Gorb S. (2009). Contact mechanics of pad of grasshopper (Insecta: ORTHOPTERA) by finite element methods. *Chinese Science Bulletin*.

[20] Wang L., Cheung J. T., Pu F., Zhang M., Fan Y. (2011). Why do woodpeckers resist head impact injury: a biomechanical investigation. *PLoS One*.

[21] Schwab I. R. (2002). Cure for a headache. *British Journal of Ophthalmology*.

[22] Yoon S. H., Park S. (2011). A mechanical analysis of woodpecker drumming and its application to shock-absorbing systems. *Bioinspiration & Biomimetics*.

[23] Kraus J. F., McArthur D. L. (1996). Epidemiologic aspects of brain injury. *Neurologic Clinics*.

[24] Mcelhaney J. H., Fogle J. L., Melvin J. W., Haynes R. R., Roberts V. L., Alem N. M. (1970). Mechanical properties of cranial bone. *Journal of Biomechanics*.

[25] Stefan U., Michael B., Werner S. (2010). Effects of three different preservation methods on the mechanical properties of human and bovine cortical bone. *Bone*.

[26] Liu S. Y., Zhang P., Lü H., Zhang C. W., Xia Q. (2012). Fabrication of high aspect ratio microfiber arrays that mimic gecko foot hairs. *Chinese Science Bulletin*.

